# A broad spectrum of posterior reversible encephalopathy syndrome - a case series with clinical and paraclinical characterisation, and histopathological findings

**DOI:** 10.1186/s12883-021-02408-0

**Published:** 2021-10-06

**Authors:** Fatme Seval Ismail, Johannes van de Nes, Ilka Kleffner

**Affiliations:** 1 Department of Neurology, University Hospital Knappschaftskrankenhaus, Ruhr University Bochum, In der Schornau 23-25, 44892 Bochum, Germany; 2 Institute of Pathology, Ruhr University Bochum, Bochum, Germany

**Keywords:** Posterior reversible encephalopathy syndrome (PRES), Reversibility, Histopathological findings

## Abstract

**Background:**

Posterior reversible encephalopathy syndrome (PRES) is clinical-neuroradiologically defined and potentially reversible, so there are limited data about histopathological findings. We aimed to describe the clinical and paraclinical features of patients with PRES with regard to its reversibility.

**Methods:**

This retrospective case series encompasses 15 PRES cases out of 1300 evaluated patients from a single German center between January 1, 2010, and June 15, 2020. PRES was established according to the diagnostic criteria as proposed by the Berlin PRES Study 2012. One of the cases studied was subject to brain autopsy.

**Results:**

From the 15 patients studied (median age 53 years, range 17–73; 11 female), 67 % presented with epileptic seizures, 40 % suffered from encephalopathy with reduced consciousness and 53 % developed delirium, while 47 % had headache and visual disturbances. Subcortical brain MRI abnormalities related to PRES were observed in all patients. One patient developed spinal ischemia and another Guillain-Barré syndrome in addition to PRES. Hypertensive blood pressure was the main underlying/trigger condition in all patients. Clinical symptoms and MRI changes were not reversible in 42 %, even progressive in 3 out of these 5 patients. Median time from symptom onset to diagnosis in these non-reversible cases was 7 days (range 0–13), while the median delay in diagnosis in the reversible group was 1 day (range 0–3). Cerebellar/brain stem involvement and status epilepticus were more frequently in patients with non-reversible disease course. Mortality due to PRES occurred in 13 % of these patients. Neuropathological examination of the brain of a 57-year-old female patient revealed major leukencephalopathic changes, fibrinoid necrosis of endothelial cells and fresh petechial hemorrhages in accordance with PRES.

**Conclusions:**

Our case series demonstrates that PRES was not reversible in 42 % of the studied patients. Delay in diagnosis seems to contribute to limited reversibility and poor outcome.

## Background

Posterior reversible encephalopathy syndrome (PRES) is clinical-neuroradiologically defined and potentially reversible [1]. It is pathogenetically characterized by acute cerebral endotheliopathy with failure of the cerebral autoregulation and disruption of the blood-brain barrier leading to vasogenic edema, mostly associated with hypertension [1, 2]. Cytotoxic edema is reported in a subset of PRES patients [3]. The clinical syndrome of PRES may show a variable spectrum of symptoms that usually occur monophasically, but recurrence in rare cases has been described [4]. Atypical distribution patterns of edema in neuroimaging are possible. Because of a low mortality with 3–6 % [5], the numbers of studies in the literature with histological findings are limited [2, 6, 7]. Many aspects of this syndrome and the pathogenetic mechanism underlying the development of PRES are as yet incompletely understood [8].

The aim of the study presented here was to describe the spectrum of clinical and paraclinical features (including MRI, CSF and EEG) of patients with PRES with regard to its reversibility. In addition, we report on the neuropathological changes in the brain of a 57-year-old female PRES patient.

## Methods

In this retrospective case series, we identified 15 patients from a total of 1300 evaluated patients using the International Classification of Diseases 10th Revision (ICD-10) diagnostic coding (G93.4, G93.6, I67.4, I67.88) in a single German neurologic center between January 1, 2010, and June 15, 2020. The included 15 patients carried the clinical-neuroradiological diagnosis of PRES according to the diagnostic criteria as proposed by Berlin PRES Study 2012 [1], one of them confirmed on histological examination at autopsy:

1. Acute development of clinical signs and symptoms typical for PRES, i.e., epileptic seizures, visual abnormalities, headaches, nausea or vomiting and other focal deficits.
2. Imaging signs typical of PRES, i.e., foci of vasogenic edema in variable distribution and severity with or without foci of restricted diffusion or hemorrhagic manifestations.
3. Presence of often multiple, toxic associations with possible endotheliotoxic effects and/or arterial hypertension (mild to severe degree).
4. Alternative causes were excluded.

Information on demographic data, clinical presentation, co-morbidities, brain MRI and CT findings, CSF analysis data, EEG findings, anticonvulsive treatment, intensive care treatment, intravenous blood pressure medication as well as maximal systolic blood pressure was obtained from the medical records. Follow-up imaging and clinical information were evaluated for reversibility, graded as complete, incomplete or progressive, while outcome was determined by functional status, as quantified by use of the modified Rankin Scale (mRS) (0–6) at admission and at discharge. In addition, brain autopsy was performed on one of the patients studied.

All procedures/protocols were in accordance with the ethic guidelines of the Ruhr University Bochum. All methods were carried out in accordance with relevant guidelines and regulations. According to legislative regulations in Germany, North Rhine-Westphalia and after consultation of the Institutional Review Board, Medical Faculty of the Ruhr University Bochum, the need for ethical approval and consent is deemed unnecessary for this retrospective case series with data collected during routine clinical work. Approval for brain autopsy was obtained.

## Results

### Clinical and paraclinical findings

Fifteen patients were identified as clinico-radiologically consistent with PRES, 7 with reversibility (#1–7), 5 with a non-reversible outcome (#8–12) and 3 with lack of data on reversibility due to loss of follow-up after referral to another hospital (#13-15). For detailed information on clinical findings, see Table 1. Eleven patients were female (73 %), median age of all patients was 53 years (range 17–73). The most common neurological symptoms were epileptic seizures occurring in 10 patients (67 %). Four out of these patients also developed status epilepticus, two patients with convulsive status, one patient with convulsive and later non-convulsive status, and another one with non-convulsive status. Impaired vision was reported in 7 patients (47 %), including one with bilateral loss of vision and another one with cortical blindness. Six patients (40 %) suffered from encephalopathy with reduced consciousness, and 6 patients (40 %) developed delirium, while 7 had headache. Mnestic deficits were described in 1 patient, focal neurological deficits (paraplegia, cranial nerve palsy) were found in 2 patients. One patient with myelodysplastic syndrome and secondary acute myeloid leukemia developed PRES and additionally Guillain-Barré syndrome after chemotherapy with allogeneic stem cell transplantation (#2), another patient with a previous medical history of prostate adenocarcinoma and multiple myeloma developed spinal ischemia in the course of PRES (#6).

**Table 1:** Main clinical and MRI features, treatment and outcome of 15 patients with PRES with regard to its reversibility

Patient, sex, age at onset in years	Underlying conditions/disease trigger	Main clinical features	Time (in days) from symptom onset to diagnosis	mRS (0–6) at admission	Brain MRI	Treatment (1) anticonvulsive medication (2) intravenous blood pressure medication	Outcome (mRS 0–6 at discharge)	Clinical and radiological reversibility
**Reversible**								
** #1, F, 17**	Arterial hypertension, coproporphyria	Epileptic seizure, impaired vision bilateral	2	2	T2/FLAIR hypersignal with diffusion impairment bi-parieto-temporo-occipital, centrum semiovale bilateral frontal	(1) Levetiracetam	0	Complete
** #2, F, 57**	Arterial hypertension, polychemotherapy, immunsuppressiva (anti-thymocyte globulin (ATG), mycophenolate mofetil (MMF), cyclosporine A (CsA), allogeneic stem cell transplantation	Headache, delirium, impaired vision; in addition to PRES Guillain-Barré syndrome	0	3	T2 hypersignal bi-parieto-occipital and bi-cerebellar, diffusion impairment cortical bi-occipital, contrast enhancement	(2) Urapidil	6 (due to septic shock)	Complete
** #3, F, 36**	Eclampsia in the puerperium (condition after sectio 3 days ago), hypertensive crisis	Epileptic seizure, headache	2	2	T2/FLAIR hypersignal (white matter) biparieto-occipital, right frontal and lateral of left basal ganglia; sulcal subarachnoidal hemorrhage left temporal	(2) Urapidil, dihydralazine	0	Complete
** #4, F, 53**	Arterial hypertension, immunsuppressiva (tacrolimus), condition after kidney transplantation	Headache, impaired vision, epileptic seizures, delirium, impaired consciousness	1	4	T2 hypersignal (juxtacortical) bi-fronto-parietal, intracerebral hemorrhage with edema bi-occipital (right > left), sulcal subarachnoidal hemorrhage right temporo-parietal and insular	(1) Levetiracetam, phenytoine (2) Clonidine, dihydralazine	3	Incomplete
** #5, M, 29**	Arterial hypertension	Headache, loss of vision both sides	3	4	T2 hypersignal subcortical bi-occipital and left temporo-parietal	(1) Levetiracetam	2	Incomplete
** #6, M, 72**	Arterial hypertension	Epileptic seizure, confusion, impaired vision, impaired consciousness	0	4	FLAIR hypersignal (cortical and subcortical) bi-temporo-occipital, hemorrhagic transformation, intrasulcal subarachnoidal hemorrhage bi-temporo-occipital; spinal ischemia (A. spinalis anterior, thoracic T8 extending to the conus)	(1) Levetiracetam (2) Urapidil	4	Incomplete
** #7, M, 66**	Serotonin syndrome due to venlafaxine with hypertension, tachykardia and fever	delirium, hallucinations, impaired consciousness	1	5	T2/FLAIR hypersignal bi-cerebellar, bi-parieto-temporal, left frontal, diffusion impairment right post-central and right cerebellar; contrast enhancement of the meninges	(1) Levetiracetam (2) Urapidil, metoprolol	3	Incomplete
**Non-Reversible**								
** #8, F, 39**	Arterial hypertension	Impaired vision bilateral, fundus hypertonicus	13	2	T2 hypersignal subcortical bihemispheric, more temporomesial, bi-cerebellar, in pons, medulla oblongata, contrast enhancement	(2) Urapidil, dihydralazine, clonidine	1	No
** #9, F, 34**	Arterial hypertension, end-stage renal failure with bilateral nephrectomy and dialysis	Headache, cortical blindness, epileptic seizures with convulsive focal status epilepticus	0	4	T2/FLAIR hypersignal bi-parieto-occipital, cortical and subcortical, diffusion lesions bihemispheric (bi-occipital, right temporal, left frontal)	(1) Levetiracetam (2) Urapidil, clonidine, dihydralazine	4	No
** #10, F, 54**	Arterial hypertension	Impaired consciousness, anisocoria, facial nerve paralysis left, gaze paresis	7	2	T2 hypersignal subcortical bihemispheric, diffusion impairment bi-cerebellar, in pons, medulla oblongata and basal ganglia bilateral, contrast enhancement in the capsula interna right	(2) Urapidil, dihydralazine	5	Progressive
** #11, F, 57**	Arterial hypertension; hypertensive encephalopathy (PRES) in the history 5 years ago	Headache, nausea, vomiting, generalized epileptic seizures with convulsive status epilepticus, mnestic deficits, agitation, impaired consciousness	7	3	Extensive symmetric and bihemispheric diffusion impairment (frontal, parietooccipital, cerebellar, temporal) with T2/FLAIR hypersignal (subcortical and cortical)	(1) Levetiracetam, valproate	6 (due to PRES)	Progressive
** #12, M, 71**	Arterial hypertension	Epileptic seizures with convulsive and later non-convulsive status epilepticus, delirium	10	3	T2 hypersignal (subcortical and cortical) bi-occipital, bi-cerebellar, corpus callosum, mesencephalon	(1) Levetiracetam, valproate, lacosamide (2) Urapidil	6 (due to PRES)	Progressive
**Not-available**								
** #13, F, 73**	Arterial hypertension	Epileptic seizures, delirium	1	4	Extensive bihemispheric white matter edema (right frontal, right parietooccipital, left occipital) with T2/FLAIR hypersignal (subcortical), small diffusion impairment right temporal	(1) Levetiracetam, lacosamide (2) Urapidil, clonidine	3	Transfer to another hospital
** #14, F, 29**	Arterial hypertension, eclampsia with emergency Caesarean section	Headache, epileptic seizures	2	2	T2/FLAIR hypersignal (subcortical) bi-occipital	(2) Urapidil	0	Transfer to another hospital
** #15, F, 48**	Arterial hypertension	Delirium, epileptic seizures with non-convulsive status epilepticus, impaired consciousness	2	3	T2 hypersignal subcortical and cortical (right > left) parieto-occipital, right thalamus	(1) Levetiracetam	1	Control MRI in outpatient radiology

*FLAIR* Fluid-attenuated inversion recovery, *mRS* Modified Rankin Scale, *PRES* Posterior reversible encephalopathy syndrome

Brain MRIs were available from all patients. Subcortical MRI abnormalities with T2-/FLAIR-hyperintense signal related to PRES were detected in order of decreasing numbers in parieto-occipital lobes (100 %), in temporal lobe and cerebellar hemisphere (each 47 %), in frontal lobe (40 %), brainstem (20 %), basal ganglia (13 %) and in thalamus (7 %). Bilateral involvement was observed in all cases, cortical involvement was described in 47 %. Foci of restricted diffusion either in the white matter or in the cortex occurred in 47 %. Four patients showed contrast enhancement, while 3 patients displayed an intrasulcal subarachnoidal hemorrhage, one of these with an additional intracerebral bleeding. In one patient PET-CT scanning revealed no uptake in brain lesions. For detailed information on neuroradiological findings, see Table 1. In our patients, there was no evidence of vasoconstriction on vascular imaging including MR-angiography, CT-angiography, digital subtraction angiography (DSA) and extra- and transcranial Doppler and Duplex sonography of brain-supplying arteries. MR-angiography alone was performed in *n* = 4 patients, in combination with CT-angiography in *n* = 1 patient, with Doppler/Duplex sonography in *n* = 3 patients, with CT-angiography and Doppler/Duplex sonography in *n* = 2 patients. Two patients have received only CT-angiography and one in combination with Doppler/Duplex sonography. DSA was performed in *n* = 2 patients.

Cerebral spinal fluid (CSF) analysis was performed in 9 patients (60 %). CSF pleocytosis (median 133 cells/µl, range 15–209) was found in 3/9 patients (33 %) (#4, 7, 10) with severe disease course of PRES, while elevated protein (median 104, range 69–261 mg/dl) was measured in 5 patients (56 %) (#4, 7–8, 12, 15). Infectious pathogens in CSF were not detected.

EEG was performed in all but one PRES patient (93 %). Epileptiform discharges were detected in 7 patients (50 %), focal slowing in 7 (50 %) and diffuse slowing in 5 patients (36 %).

Underlying conditions and trigger of PRES:

Hypertensive blood pressure was measured at hospital admission in all patients. The maximum systolic blood pressure ranged from 155 to 275 mmHg (median 196 mmHg). Underlying conditions triggering or contributing to PRES were coproporphyria (#1), immunosuppressive treatment (tacrolimus (#4); anti-thymocyte globulin, mycophenolate mofetil, cyclosporine A (#2)), chemotherapy with allogeneic stem cell transplantation (#2), eclampsia during pregnancy/puerperium (#3 and #14, respectively), serotonine syndrome due to venlafaxine (#7), history of PRES (#11) (Table 1).

Comorbidities included tumor (prostate adenocarcinoma *n* = 1, multiple myeloma *n* = 1, myelodysplastic syndrome with secondary acute myeloid leukemia *n* = 1), type 2 diabetes (*n* = 3), hypertensive nephropathy with glomerular proteinuria (*n* = 1), chronic renal insufficiency (*n* = 1), condition after kidney transplantation (*n* = 1), condition after bilateral nephrectomy with dialysis (*n* = 1), hepatitis of unknown origin (*n* = 1), alcohol dependency (*n* = 3), sepsis (*n* = 1), psychosis (*n* = 1), history of epilepsy due to tuberous sclerosis complex (*n* = 1), and vascular dementia (*n* = 1).

### Treatment

Anticonvulsive treatment with levetiracetam was administered in 10 patients. Intensive care treatment was indicated in 12 cases (80 %) with median duration of 5 days (range 2–35). Intravenous blood pressure medication as monotherapy or combination (urapidil, clonidine, dihydralazine) was administered in 11 patients (73 %). In one patient with fatal course, corticosteroids were administered before admission possibly contributing to the worsening of PRES due to increased risk of acute hypertension.

### Reversibility and outcome

Complete clinical and brain imaging reversibility was reported in 3 patients (25 %) (#1–3), incomplete in 4 patients (33 %) (#4–7). Clinical symptoms and MRI changes were not reversible or even progressive in 5 patients (42 %) (#8–12) (Table 1). Duration of follow-up in the 12 patients for whom this was available was median 18,5 days (range 7-141). In the group with non-reversible disease course, median time from symptom onset to diagnosis was 7 days (range 0–13), thus generally longer compared to the group of patients with reversible course (1 day, range 0–3). Cerebellar and brain stem involvement in brain MRI was observed in 4 out of 5 patients with a non-reversible course (80 %) and in 2 out of 7 patients with reversible course (29 %). Status epilepticus was observed only in patients with non-reversible disease course.

Median mRS at admission was 3 (range 2–5) and at discharge 3 (range 0–6). Two patients (13 %) died of PRES, one of those patients with a fatal course was admitted to our department because of status epilepticus and massive brain edema (#12).

### Case #11

#### Clinical presentation and neuroradiological examination

**Fig. 1:**
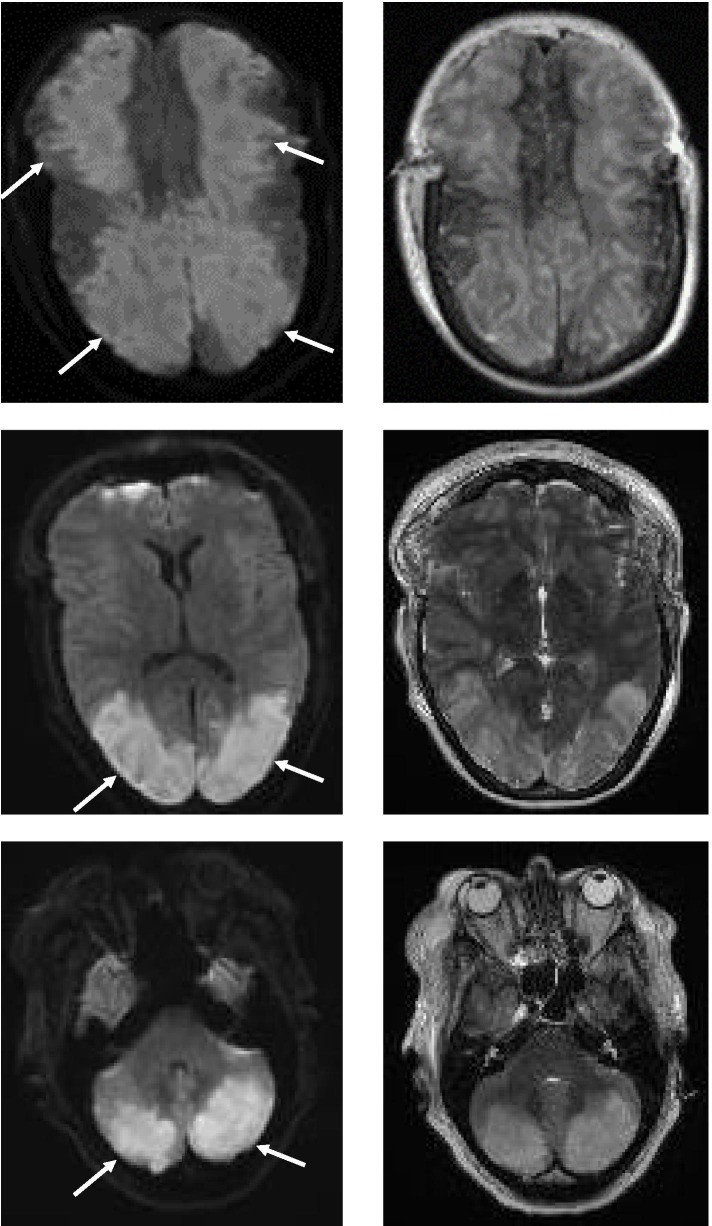
Brain MRI (transversal sequences) in a fatal case of PRES due to hypertension. Bilateral, symmetric and extensive diffusion restrictions (on diffusion-weighted imaging (DWI)) (*white arrows*) (left panels) and T2 hyperintense signal (right panels) due to full-blown manifestation of PRES with following bifrontal craniotomy

The second patient with a fatal course, a 57-year-old woman (#11), was known for diabetes type 2 and hypertensive encephalopathy (PRES) in the previous medical history 5 years before. She was referred from another clinic to our hospital for a bifrontal craniotomy because of rapidly progressive generalized brain edema. Immunotherapy including intravenous methylprednisolone for 5 days was carried out for a suspected autoimmune encephalitis in the other clinic before transfer to our hospital. Post-operative MRI revealed bilateral symmetric and extensive diffuse restriction with T2 hyperintense signaling (frontal, parietooccipital, cerebellar, temporal) as full-blown manifestation of PRES (Fig. 1). Despite intensive medical treatment, the patient eventually died due to supra- and infratentorial herniation. Approval for brain autopsy was obtained.

#### Macroscopic examination

The brain (1.500 g) showed a generalized edema with flattened brain surface, bilateral uncal and cerebellar tonsillar herniation with major infarctions in the occipital lobes due to compression of the posterior cerebral arteries and in the cerebellar hemispheres due to compression of the superior cerebellar arteries. In addition, a major bifrontal extracalvarian herniation (fungus cerebri) related to bifrontal craniotomy as neurosurgical decompression therapy and major bifrontal infarctions were present (not shown).

#### Histopathological examination

**Fig. 2:**
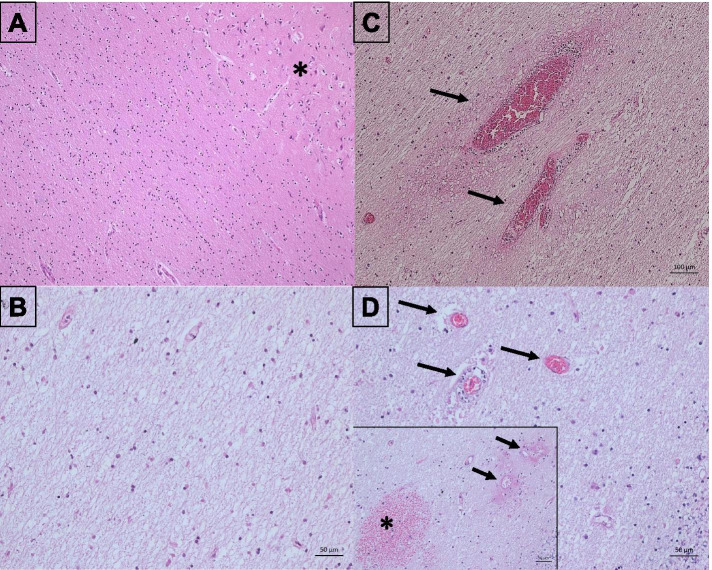
Histopathological findings in a fatal case of PRES due to hypertension. **A** The frontal basal white matter appears normal. The *black asterisk* at the top right is to identify cerebral cortex with fresh hypoxemic-ischemic nerve cell damage in the form of cytoplasmic shrinkage, hypereosinophilia and nuclear condensation. **B** In contrast to frontal basal (**A**), the slices obtained from occipital white matter are characterized by diffuse myelin pallor and mildly increased glial cell density. **C-D** Slices obtained from cerebellar white matter with myelin pallor and mild reactive glial cell proliferation. In panel **C**, one sees two thin-walled blood vessels with hyperemia and a perivascular exsudate indicative of endothelial cell damage (*black arrows*). Panel **D** shows three thin-walled hyperemic blood vessels (*black arrows*). The central blood vessel is surrounded by some macrophages with vacuolated cytoplasms (“phagocytes”) indicative of myelin sheath break down products. In the small slice embedded in panel **D**, two thin-walled blood vessels (*black arrows*) with fibrinoid vascular wall necrosis can be seen at the top right and a petechial microbleeding area (*black asterisk*) at the bottom left. Hematoxylin and eosin staining of all slices; Scale bar = 100 μm/50 µm

Tissue specimens displayed extensive leukencephalopathic changes in a major part of the cerebral and cerebellar hemispheres, viz., diffuse myelin pallor and mild astroglial activation, some thin-walled blood vessels with fibrinoid wall necrosis and minor fresh petechial hemorrhages. In addition, a few macrophages with vacuolated cytoplasm around some blood vessels were observed, indicative of phagocytosis of myelin sheath break down products. The cerebral and cerebellar cortex, basal ganglia and brain stem nuclei showed major hypoxic-ischaemic neuronal changes (Fig. 2A-D).

## Discussion

In this retrospective study on 15 patients with PRES, hypertensive blood pressure from mild to severe degree was detected in all patients, supporting the assumed association of hypertension with the development of PRES. Edematous bilateral lesions were encountered in the occipital and parietal lobes of all patients studied, while additional cerebellar, temporal and frontal lobe involvement occurred in about 50 % of the cases. Involvement of other brain regions was also observed, viz., brain stem (20 %), basal ganglia (13 %) or thalamus (7 %). Hemorrhage and contrast enhancement occurred in some cases (27 %). The most common neurological symptoms were seizures. Visual disturbances, encephalopathy, delirium and headache represented other common symptoms. These findings are consistent with other study results [1, 5, 9, 10].

Interestingly, in our case series irreversibility was detected in 42 % of patients in contrast to 18 % in the Berlin PRES Study 2012 [1] and to 10–25 % in the literature [5]. However, residual structural lesions including focal gliosis, infarction, posthemorrhagic residua, atrophy and laminar necrosis were seen in 43 % of patients in the Berlin PRES Study [1]. There are some arguments that can explain the higher percentage of cases with non-reversible disease course (42 %) in our study. First, poor outcome of PRES seems related to a delayed diagnosis, thus supporting a previously report on delay in the elimination of the causes as an important predictor of poor outcome [11]. In addition, overrepresentation of critically ill patients (80 % of our patients required intensive care treatment) may be a potential indication of an irreversible course of PRES. Further, we followed the criteria proposed in the Berlin PRES Study 2012 for inclusion of the patients [1]. However, reversibility is not included in these criteria, because PRES is not always reversible and residual lesions may be encountered more frequently than commonly expected [1, 5].

In our study, PRES occurred in a wide range of disorders and predisposing conditions ranging from hypertension, eclampsia to stem cell transplantation, exposure to various immunosuppressants and cytostatic drugs as well as coproporphyria and serotonin syndrome. During the recent coronavirus disease 2019 (COVID-19) pandemic, COVID-19 infection has been identified as a further co-morbid condition of PRES [12–14]. In one case, PRES showed a recurrence after a first episode 5 years before, suggesting a certain predisposition and the risk of recurrence if trigger factors are not eliminated, in this case hypertension. PRES can even involve the spinal cord, one patient with PRES in our study suffered in addition to brain lesions from spinal ischemia [2]. In this case, the spinal ischemia could be caused by PRES. In another case with Guillain-Barré syndrome in addition to PRES, we assume that the diseases occurred independently of each other, since PRES and Guillain-Barré syndrome have different pathomechanisms. CSF pleocytosis was found in 33 % and elevated protein in 56 % of our patients. We assumed the CSF pleocytosis as a result of a massive dysfunction of the blood brain barrier due to severe PRES, leading to increase of cell count following blood leakage. In two of these patients, subarachnoidal hemorrhage was detected. Other causes for CSF pleocytosis such as infection were excluded. In a previous study, all patients with CSF pleocytosis had atypical imaging features such as infarction or subarachnoidal hemorrhage [15]. In the literature, elevated total protein in CSF was reported to be correlated with severity of edema in PRES patients, whereas pleocytosis was rare [16], supporting the theory of a dysfunctional blood brain barrier.

Previous studies reported that vasoconstriction occurs in about 15–30 % of patients with PRES who undergo angiography. Otherwise, PRES has also been reported in 17–38 % of patients with reversible cerebral vasoconstriction syndrome (RCVS) [5]. Since pathophysiological mechanisms of PRES and RCVS are not completely known, it is still subject of debate whether PRES and RCVS are independent syndromes and sometimes overlap or are part of a continuum process [17]. In our patients, there was no evidence of vasoconstriction on vascular imaging including MR-angiography, CT-angiography, digital subtraction angiography (DSA) and extra- and transcranial Doppler and Duplex sonography of brain-supplying arteries.

In our study population, mortality due to PRES was with 13 % higher than the estimated 3–6 % in hospital-based retrospective studies [5], indicating that early diagnosis, management of increased blood pressure and elimination of trigger factors are crucial for the outcome. However, another single center study reported a fatal outcome of 19,1 % in their patients [18]. The histopathological findings in our patient with fatal course was consistent with previous findings [2, 6, 7] which confirm appearance of vasogenic and cytotoxic edema. Major histopathological findings included myelin pallor, prominent white matter vacuolization, swollen vascular endothelium, petechial hemorrhages, gliosis [2].

Limitation of our study is the small number of included patients and the retrospective nature. Further studies with a larger number of patients and a prospective study design are needed to confirm the cause-effect conclusions.

## Conclusions

Our case series demonstrates a broad spectrum of clinical and neuroradiological manifestations of PRES, which was not in all cases reversible. Delay in diagnosis seems to contribute to limited reversibility and poor outcome. Histopathological findings in PRES are rare and can contribute to a better understanding of the pathophysiology.

## Data Availability

All data generated or analysed during this study are included in this published article. The data are available from the corresponding author upon request. No specific database was used for this case series, so public access to the database(s) is not applicable.

## Abbreviations

CSF: Cerebral spinal fluid
EEG: Electroencephalography
mRS: modified Rankin Scale
PET-CT: Positron emission tomography–computed tomography
PRES: Posterior reversible encephalopathy syndrome
RCVS: Reversible cerebral vasoconstriction syndrome
